# Quality of life and clinical factors in women with endometriosis, the role of dienogest vs EE/dienogest over time: a single-center study

**DOI:** 10.1007/s00404-023-06942-9

**Published:** 2023-02-04

**Authors:** Antonio Maiorana, Pietro Alfano, Antonella Mercurio, Salvatore Marcantonio, Gabriella Minneci, Domenico Incandela, Palma Audino

**Affiliations:** 1 Gynaecologic and Obstetric Unit, ARNAS Civico Di Cristina Fatebenefratelli, Piazza Nicola Leotta 4, 90127 Palermo, Italy; 2 Institute of Translational Pharmacology (IFT), National Research Council of Italy, Via Fosso del Cavaliere 100, 00133 Rome, Italy; 3 Quality, Planning and Strategic Support Area, University of Palermo, Piazza Marina 61, 90133 Palermo, Italy; 4 Psychology Unit, ASP Palermo, Via Roma 519, 90139 Palermo, Italy

**Keywords:** Dyspareunia, Health-Related Quality of life, Sexual satisfaction, Pain

## Abstract

**Purpose:**

The aims of this observational study were: to assess the relationship between psychological variables, pain, Duration Untreated Endometriosis (DUE) in a sample of women with Endometriosis; and to assess the effect of dienogest 2 mg/daily (DNG) and dienogest/ethinylestradiol 0.03 mg/daily (EE/DNG) on Symptoms, QoL, HRQoL, pain and sexual satisfaction, over time.

**Methods:**

64 women constituted the study group; (56%) took DNG and (44%) took EE/DNG. VAS, SF-36, EHP-30 and ISS were used to assess endometriosis-associated pelvic pain, QoL, HRQoL and sexual satisfaction, respectively. The study included one follow-up at 18 months.

**Results:**

At T0, a longer period of DUE was related both to worst HRQoL and Physical QoL. At T1, a correlation was found between longer DUE and worst HRQoL. At T0, a negative correlation was found between VAS and PCS and between VAS and EHP-30. At T1, the same above correlation was found between VAS and PCS/MCS and VAS and EHP-30 scale. There was a correlation between ISS and VAS. ANOVA showed a reduction in dysmenorrhea, in general pain level and an improvement in emotional wellbeing, relationship with medical profession, and PCS over time, regardless to type of treatment. Moreover, a significant time × treatment group interaction for dysmenorrhea was found.

**Conclusion:**

DUE and pain are important variables related to psychological aspects of women with endometriosis. Treatment with both DNG and EE/DNG may have positive effects on the QoL, HRQoL and symptoms. Moreover, DNG seems to have a greater effect than EE/DNG on dyspareunia reduction over time.

## What does this study add to the clinical work

**Table:** 

The results of this study highlight that diagnostic delay and pain are related to worsening of different component of both QoL and HRQoL of women with endometriosis. Moreover, DNG seems to have a greater effect than EE/DNG on dyspareunia reduction over time. Improvement in pain symptoms and QoL is the central goal in the treatment of endometriosis toward a global management of the disease.	

## Introduction

Endometriosis is a chronic non-malignant estrogen-dependent condition characterized by the presence of endometrial-like tissue outside the uterus affecting 10% of women of reproductive age [1] including very young girls, from all ethnic and social groups [2]. The causes of endometriosis have not yet been determined [3]. Endometriosis is associated with symptoms of dysmenorrhea, chronic pelvic pain, dyspareunia, dyschezia [4], up to 50% of women with endometriosis experience infertility [5] with an impairment of QoL [6]. Recent studies consider endometriosis as a disabling condition that may affect the social relationships, mental health, and sexual activity of women [7, 8]. Despite its considerable impact, endometriosis is often under-diagnosed; an Italian study suggests that only 6 out of 10 cases in the general population are diagnosed [9]. There are several factors that can complicate the diagnosis: asymptomatic cases, the late appearance of symptoms and the increased presence of comorbidities with similar symptoms to endometriosis. Symptoms begin during adolescence; thus, treatment is often started several years after use of NSAIDs [10], medical and psychosocial factors contribute to a delayed diagnosis [11]. In this study, we consider the period between the onset of endometriosis symptoms and diagnosis as “Duration Untreated Endometriosis (DUE)”. Among current management approaches, surgery and hormonal drugs are considered as primary treatment to reduce recurrences and to improve the QoL in women suffering from endometriosis [12]. However, medical treatment may be not a permanent solution for symptoms and infertility, so chronic pelvic pain and related symptoms can affect women throughout their fertile life. Pain and infertility may have negative effect on QoL for patients with endometriosis [13]. Škegro et al. showed that higher pain level was related to poorer quality of life [14], emphasizing both physical and psychological aspect of the disease also for treatment project.

A key factor correlated with disease and QoL is the available treatment method. This is one of the most crucial aspects for both physicians and patients, so major consideration should be given not only to efficacy but also to the long-term safety and tolerability of the treatment options that are available [1]. DNG is a synthetic progestin that is currently used for clinical treatment of endometriosis with a dose of 2 mg daily [15], it is an effective and well-tolerated treatment for endometriosis-related pain [16]. It is know that patients are willing to accept the spotting that DNG can cause given the pain relief experienced [17] and may also have positive effects on their QoL and sexual life [18].

Moreover, a reduction of pain was observed in patients using a combination of ethinylestradiol and dienogest in continuous regimen [19] as well as with dienogest alone [17].

Aims of the present study:

- To assess the relationship between psychological variables, pain, Duration Untreated Endometriosis (DUE) in a sample of women with endometriosis.
- To assess the 18-month effect of dienogest 2 mg/daily (DNG) and dienogest/ethinylestradiol 0.03 mg/daily (EE/DNG) on symptoms, QoL, HRQoL, pain and sexual satisfaction in women with endometriosis over time.

### *Hypothesis*

The psychological state of women affected by endometriosis is related to symptoms and to the duration of the untreated disease. The response of symptoms to DNG could be better than the response to EE/DNG.

## Materials and methods

This observational, single-center study was conducted on a sample of women with endometriosis referred to a center for diagnosis and treatment of endometriosis with dedicated physicians, psychologists, and nurses, of the Department of Obstetrics and Gynecology at the ARNAS Civico Hospital in Palermo, in Southern Italy, between October 2019 and June 2021.

### Participants

85 women with endometriosis were consecutively recruited. All patients received personalized clinical indications regarding the use of medical therapy with DNG and EE/DNG administered orally. Side effects were collected during routine clinical practice, two participants reported vaginal bleeding and loss of libido were excluded from the final data analysis because they spontaneously suspended their therapy. Nineteen participants were excluded because of missing data.

Finally, 64 women completed all study procedures, 36 (56%) took DNG and 28 (44%) took EE/DNG. These women reported that treatment was well tolerated and that the therapy wellness was superior to the side effects.

**Fig. 1:**
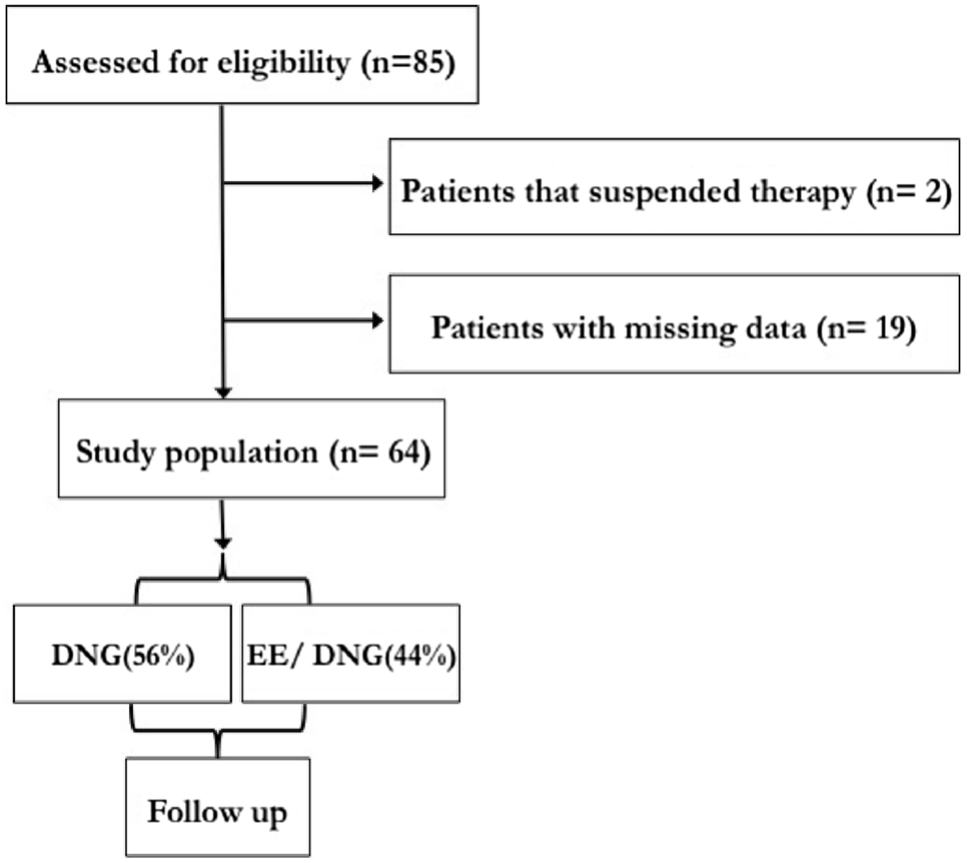
Flow chart of the study

According to routine practice, women came back for clinical assessment and psychological evaluation after 18 months (T1), and completed all measures at both Time 0 (T0) and Time 1 (T1), (Fig. 1). Some women (50%) performed the T1 procedures a few weeks after the visit scheduled at 18 months because of patients’ specific clinical needs; this variability was taken into account in the analysis as a covariate.

All subjects gave written informed consent. The individual privacy of clinical data was guaranteed under Italian law. Eligible for the study were women aged 18 years or more not seeking pregnancy, with a surgical diagnosis or a clinical/instrumental diagnosis of endometriosis, who had been taking DNG or EE/DNG at the time of recruitment.

This study has been conducted according to the Declaration of Helsinki for studies involving patients and humans and was approved by the Local Institutional Ethics Committee (N° 18/14 Feb 2018).

### Procedures

#### Health related Quality of Life Short Form Survey “SF-36”

The questionnaire consisted of 36 items, and transformed to give eight summary scales measuring health concepts, summarized in two component factors: physical health (PCS) and mental health (MCS). The raw scores were converted into standard scores (*M* = 50 ± 10), range 0–100, in accordance with the questionnaire guidelines [20].

#### Health-Related Quality of Life (HRQoL) endometriosis health profile 30 “EHP-30”

This is a disease-specific tool to evaluate the HRQoL in women suffering from endometriosis. The EHP-30 is composed of two parts: a core questionnaire containing five scales applicable to all women with endometriosis (30 items): pain, control and powerlessness, emotional wellbeing, social support, self-image and a modular part containing six scales which do not necessarily apply to all women with endometriosis (23 items): work life, relationship with children, sexual intercourse, medical profession, treatment and infertility. The score ranges from 0 (best possible health status) to 100 (worst possible health status) [21].

#### Index of sexual satisfaction “ISS”

This scale aims to assess problems relating to the sexual aspects of a relationship, measuring subject’s feelings about behaviors, attitudes, events, affective states and preferences associated with sexual intercourse between partners. It contains 25 questions, the obtained scores ranged from 0 to 150. A cutoff score ≥ 75, indicates a higher degree of sexual satisfaction [22].

#### Visual Analogical Scale “VAS”

The Visual Analogic Scale was used to define endometriosis-associated pain, chronic pelvic pain, dysmenorrhea, dyspareunia and dyschezia.

The instrument measures the pain level across a continuum of values, from the patient's perspective, their pain does not make discrete jumps, as a categorization of none, mild, moderate and severe would suggest. VAS is a horizontal line, 100 mm in length, anchored by word descriptors at each end. The patient marks on the line the point that they feel represents their perception of their current state. VAS has been repeatedly used in the literature as a way to measure patients’ perceived pain [23, 24].

Study variables

- dysmenorrhea, dyspareunia and dyschezia;
- presence of spotting, headache, weight gain, low libido
- age
- age at menarche
- age diagnosis
- age first symptoms
- duration Untreated Endometriosis (DUE) refers to the period (years) between the onset of endometriosis symptoms and the diagnosis
- presence of children in the household and their age
- volume of endometrioma sagittal and antero-posterior compartment

### Statistical analysis

All statistical analyses were performed using the SPSS statistical software version 20, with screening for missing data.

A tool was performed [25] to determine the minimum total sample size required for this study, and the results showed that at least 54 participants were needed to register an *α* level of 0.05 and to have 80% power.

Descriptive statistics were used to summarize the study participants’ clinical and psychological variables presented as mean (SD), median values (range) and frequency (%) as appropriate according to data distribution. The differences in patient characteristics between treatment groups (DNG and EE/DNG) were assessed with the Sample *T* Test and *χ*^2^. The psychological and clinical differences between treatment groups (DNG and EE/DNG) were performed using the Sample *T* Test and Mann–Whitney *U* Test. The correlations between chronological, clinical and psychological variables both at T0 and at T1 were performed with R Pearson. The effects of treatment over time on the clinical and psychological variables were evaluated by a 2 (treatment) × 2 (time) mixed-design analysis of variance (ANOVA). The independent variable was given by the 2 approaches to treatment: DNG and EE/DNG. The dependent variables were EHP-30, SF-36, ISS, VAS, dysmenorrhea, dyspareunia and dyschezia with checks for clinical variables that differed by group (age diagnosis, DUE and time variability). The level of significance was set at *α* = 0.05.

## Results

64 women with endometriosis who completed both time surveys were aged between 19 and 49 years old (*M* = 32.61 SD = 7.3); 36 of them (56%) took DNG and 28 (44%) took EE/DNG. The descriptive statistics summarizing the participants’ demographic and clinical characteristics in the two treatment groups and the *p* values for test statistics assessing group differences at baseline are listed in Table 1.

**Table 1:** Patient characteristics at baseline *χ*^2^, Independent Sample *T *Test, for DNG (*n* = 36) and EE/DNG (*n* = 28) Groups

	DNG No (%)	EE/DNG No (%)	*p* value^a^
Education			
Primary education	1 (2%)	1 (2%)	*NS*
Lower secondary education	14 (22%)	8 (12%)	*NS*
Upper secondary education	16 (25%)	15 (23%)	*NS*
Degree	5 (8%)	4 (6%)	*NS*
Marital status			
Single	8 (12%)	4 (6%)	*NS*
Unmarried couple	13 (20%)	14 (23%)	*NS*
Married	15 (23%)	10 (16%)	*NS*
Occupation			
Unemployed	15 (23%)	13 (20%)	*NS*
Student	3 (5%)	5 (8%)	*NS*
Housewife	4 (6%)	3 (5%)	*NS*
Employee	14 (22%)	4 (6%)	*NS*
Self-employed	0 (0%)	3 (5%)	*NS*

	Mean (SD)	Mean (SD)	*p* value^b^
Chronological Variables			
Age	34 (8.3)	31 (5.5)	*NS*
Age diagnosis	31 (8.14)	27 (5)	*0.016*
Age first symptoms	27 (9.4)	23 (6.1)	*0.037*
Age at menarche	12 (1.39)	12.14 (1.27)	*NS*
Duration untreated endometriosis	3.55 (6.18)	3.64 (5.05)	*NS*
Children			
Yes children	14 (22%)	12 (19%)	*NS*
Age of children in household	14.85 (8.53)	11.58 (6.65)	*NS*

	No (%)	No (%)	*p* value^a^
Symptoms			
Spotting	6 (9%)	7 (11%)	*NS*
Headache	2 (3%)	1 (2%)	*NS*
Weight gain	2 (3%)	1 (2%)	*NS*
Low libido	4 (6%)	2 (3%)	*NS*

*P*-values < 0.05 were classified as statistically significant

*NS* as non significant

^a^*χ*^2^

^b^Sample *T* Test

No significant group differences were found except for age of diagnosis (*t*(62) = 2.48, *p* < 0.05), and age of onset of first symptoms (*t*(62) = 2.13, *p* =  < 0.05). Side effects prevalence was not significant different in both group. Neither at T1 were found significant differences concerning side effects for two groups. The descriptive statistics for psychological and clinical variables between treatment groups at T0 and T1 are listed in Table 2. No significant differences were found for SF-36, EHP-30, ISS, VAS, dysmenorrhea, dyschezia and dyspareunia between the groups.

**Table 2:** Descriptive, Independent Sample *T *Test, *U* Mann–Whitney of psychological and clinical variables for DNG (*n* = 36) and EE/DNG (*n* = 28) Groups at T0 and T1

	T0			T1		
	DNG	EE/DNG	*p*	DNG	EE/DNG	*p*
	Mean (SD)	Mean (SD)	*T* test	Mean (SD)	Mean (SD)	*T* test
SF-36						
PCS	41.14 (10.7)	40.04 (9.6)	*NS*	49.64 (13.05)	49.44 (6.82)	*NS*
MCS	36.17 (11.4)	40.39 (11.1)	*NS*	40.86 (14.59)	41.14 (9.78)	*NS*
VAS	8.6 (1.7)	7.9 (2.5)	*NS*	3.22 (3.05)	2.7 (2.9)	*NS*
ISS	17.7 (18.9)	11.8 (11.1)	*NS*	16 (19.6)	11.1 (12.2)	*NS*
EHP-30						
Pain	52.6 (22.5)	41.6 (21.9)	0.041	16.4 (20.9)	14.6 (11)	*NS*
Control and powerlessness	44.1 (31.7)	43.4 (27.7)	*NS*	13.3 (23.1)	18.2 (23.1)	*NS*
Emotional wellbeing	45.6 (23.6)	43.5 (23.8)	*NS*	30.19 (28.5)	26.8 (20.9)	*NS*
Social support	47.8 (31.8)	57.9 (31)	*NS*	32.6 (33.9)	39.9 (35)	*NS*
Self-image	34.2 (31.8)	24.7 (31.9)	*NS*	26.9 (30.9)	15.41 (25.2)	*NS*
Work	21.7 (33.7)	13.4 (24.4)	*NS*	6.9 (16.3)	4.6 (15.6)	*NS*
Relationship with children	6.9 (20.4)	7.1 (21.4)	*NS*	1.1 (6.3)	1.4 (7.3)	*NS*
Sexual intercourse	47.4 (35.5)	45.5 (31.01)	*NS*	26.2 (31.7)	27.6 (32.1)	*NS*
Relationship with medical profession	21.4 (31.5)	23 (32.8)	*NS*	4.3 (14.5)	2.1 (9.7)	*NS*
Treatment	0 (0)	1.79 (9.5)	*NS*	6.7 (22.5)	13 (18.4)	*NS*
Infertility	28.6 (34)	21 (26.7)	*NS*	31.4 (34)	20.8 (28.7)	*NS*
Dysmenorrhea	8.29 (2.53)	6.24 (4.23)	*NS*	0 (0)	0 (0)	*NS*
Dyschezia	3.87 (4.02)	2.96 (4.09)	*NS*	1.74 (3.32)	1.40 (2.9)	*NS*
Dyspareunia	5.71 (3.87)	6 (3.89)	*NS*	3.35 (3.79)	3.04 (3.60)	*NS*

	Median (range)	Median (range)	*U* Mann Whitney	Median (range)	Median (range)	*U* Mann Whitney
Volume sagittal compartment	39 (14–77)	25.5 (0–41)	*NS*	26 (0–63)	18 (8–29)	< *0.05*
Volume antero-posterior compartment	27 (12–61)	17.5 (0–35)	*NS*	18 (0–44)	13 (5–23)	*NS*

*P*-values < 0.05 were classified as statistically significant

*NS* as non significant

At T0, one sample *T* Test found significant differences between the PCS mean values of our sample (*M* = 40.66 SD = 10.20) and the Italian female standardization sample values (*t*(63) = – 6.46, *p* = 0.000) but not at T1 (*M* = 49.64 SD = 10.70). The MCS mean values were also significantly lower than the mean for the Italian general female population both at T0 (*M* = 38.02, SD = 11.40) (*t*(63) = – 4.90, *p* = 0.000) and at T1*(M* = 40.98, SD = 12.62) (*t*(63) = – 2.54, *p* < *0.05*).

In the entire sample, no score was found for significant clinical symptoms for the 11 investigated scales of EHP-30.

No clinical scores concerning sexual problems (ISS) in relationships were evaluated both at T0 than at T1*.*

Table 3 shows the correlation between psychological variables, VAS and DUE, in all the samples.

**Table 3:** Correlation for psychological variables in all the samples at T0 and T1

	T0		T1	
	VAS	DUE	VAS	DUE
SF-36				
PCS	*r* = – 234, *p* < 0.001	*r* = – 0.263, *p* < 0.05	*r* = – 400, *p* = 0.001	
MCS			*r* = – 0.272 *p* < 0.05	
ISS			*r* = 0.428, *p* = 0.001	
EHP-30				
Pain	*r* = 0.548, *p* < 0.001	*r* = 0.289, *p* < 0.05	*r* = 0.612, *p* < 0.001	*r* = 0.277, *p* < 0.05
Control and Powerlessness	*r* = 0.411, *p* = 0.001		*r* = 0.467, *p* < 0.001	*r* = 0.338, *p* < 0.01
Emotional wellbeing	*r* = 0.377, *p* < 0.01		*r* = 0.283, *p* < 0.05	
Social support			*r* = 0.383, *p* < 0.01	*r* = 0.274, *p* < 0.05
Self-image				
Work	*r* = 0.340, *p* < 0.01	*r* = 0.350, *p* < 0.01		
Relationship with children				
Sexual intercourse	*r* = 0.313, *p* = < 0.05		*r* = 0.431, *p* = 0.001	
Relationship with medical profession		*r* = 0.336, *p* < 0.01		
Treatment	*r* = 0.258, *p* < 0.05	*r* = 0.338, *p* < 0.01	*r* = 0.258, *p* < 0.05	*r* = 0.338, *p* < 0.01
Infertility				

### ANOVA direct effect

Age of diagnosis and DUE were included as covariates in the ANOVA analyses to check the important influence of diagnosis time in the medical history of the patients. Also, the difference in time from T0 and T1 was inserted as a covariate to account for this variability. The mixed model revealed a significant main effect for dysmenorrhea over time (*F*(1, 59) = 16.27, *p* < 0.001): at the follow-up, the dysmenorrhea score was significantly lower than the dysmenorrhea score at T0 regardless of the type of treatment. The same significant main effect over time was also found for VAS: general pain level decreases over time (*F*(1,58) = 30 *p* < 0.001) for both groups treatment. EHP-30 areas also improved significantly over time, regardless of the type of treatment: emotional wellbeing *F*(1, 59) = 4.45, *p* < 0.05), and the relationship with the medical profession *F*(1, 59) = 6, *p* < 0.05). As concerns SF-36, an improvement in the physical component was found over time (*F* (1, 59) = 7.21, *p* < 0.01). No significant results were found for the mental component.

### Interaction effect

**Fig. 2:**
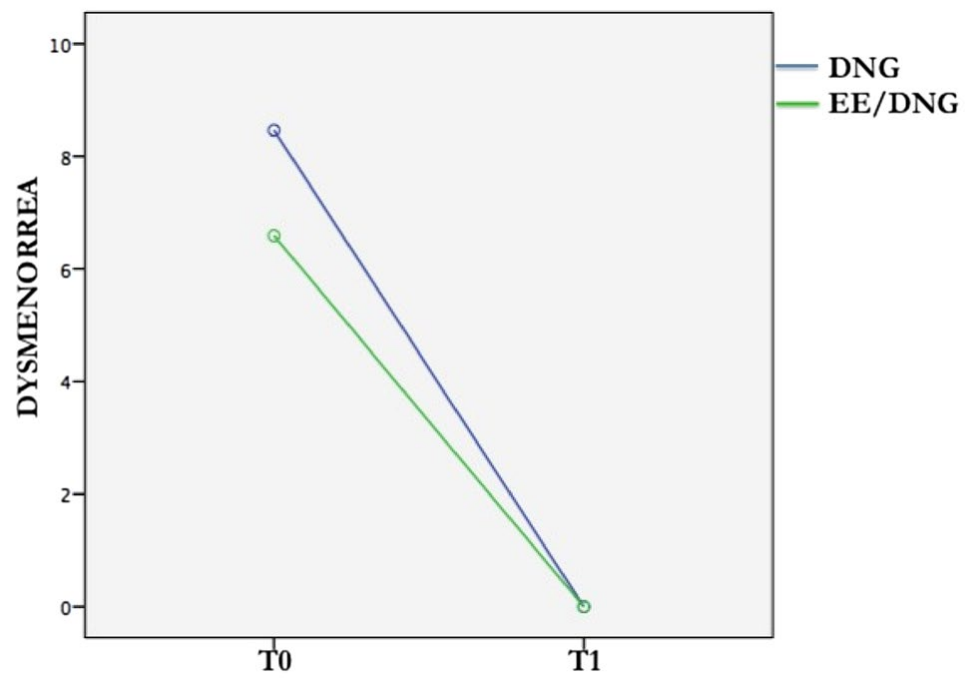
Interaction effect for time and treatment on dysmenorrhea

A mixed-design analysis of variance revealed a significant time × treatment group interaction for dysmenorrhea (*F*(1, 59) = 5.13, *p* < 0.05). More specifically, the dysmenorrhea level of women taking DNG decreased more than that of the EE/DNG group over time; the differences between groups were not constant for the two times (Fig. 2).

## Discussion

This study aimed primarily to assess the relationship between psychological variables, pain and Duration Untreated Endometriosis (DUE) in a sample of women with endometriosis. Second, to evaluate the effect of DNG and EE/DNG on symptoms, QoL, HRQoL, pain and sexual satisfaction in endometriosis patients over time, at 18 months (follow-up). The impact of endometriosis on a woman’s perception of her quality of life is substantial and wide ranging. The sample studied presented low QoL levels for both the mental and physical components compared to Italian standard population, so it represents an important indicator of a disease-related disabling state. The quality of life of women with endometriosis is influenced by many factors. At T0, women described limitations in their physical health, social and personal activities due to emotional and health problems, physical pain, loss of strength and considered their health as poor. Both at T0 and at T1 most patients reported psychological distress, social and personal limitations due to emotional and health problems.

We called the time between the onset of endometriosis symptoms and diagnosis Duration Untreated Endometriosis (DUE) analyzing the relationship with psychological variables. Diagnosis delays have been well documented: Nnoaham [26] reported an average diagnosis delay of 6.7 years, Soliman in 2017 [27] of 4.4 years and in our group; the DUE mean was reduced to 3.59. We consider that our results show both that diagnosis in an endometriosis reference center may be faster and also that, over the last years, there has been an increase of information in the medical–scientific field and hence a reduction in diagnosis time [28]. Our results are in line with the literature, which negatively considers a delay in appropriate treatment both for the QoL of a woman and for progression of endometriosis [29]. We point out that a longer DUE is related to worse HRQoL in different components, showing higher pain perception, work problems, lack of confidence in the medical profession and in treatment. Also PCS was more compromised in women with a longer DUE period: they described higher limitations with regard to physical, social and personal activities because of the disease. At T1 women with longer DUE referred greater impairment regarding pain perception, social support and symptom control and less trust in treatment than women with shorter DUE.

In line with several studies [8, 14], we confirmed the role of pain as an important factor influencing physical, psychosocial or emotional limitations. In our sample, higher pain perception is related to worse QoL as regards the physical component at T0 and both physical and mental components at T1, with more limitations in self-care, physical, social and personal activities, fatigue, distress, emotional problems and poor perception of health. So, according to Pessoa [30], it is important to consider clinical manifestation as central goals in treatment of endometriosis, not only infertility. As concerns HRQoL, we did not find significant clinical alerts of impairment in our group. However, different areas were related to pain both at T0 and at T1, meaning that women that experienced a higher pain level expressed more difficulty in management of pain and symptom controls in their daily activities, mood swings, concern about pains during sexual intercourse, and lack of confidence in treatment. Work difficulties are only related to pain at T0 and misunderstanding by others at T1. Nor, as concerns couples’ sexual problems, did we find clinical levels for all the groups, only a few people reporting significant problems in the sexual area. Couples’ sexual problems were greater when women experienced high pain levels, but only at T1. Poor satisfaction in sexual life worsens the overall quality of life and can lead to anxiety and mood symptoms [31].

As concerns the effect of DNG and EE/DNG over time, we found significant results: dysmenorrhea, VAS, emotional wellbeing, the relationship with the medical profession, and the PCS improved for both the DNG and EE/DNG groups over time. Hence, general and psychological wellbeing can increase in a setting of trust where doctor and patient cooperate to manage the symptoms successfully, regardless of the type of treatment. Moreover, we noted an interaction effect between time and treatment only for dysmenorrhea. Even if dysmenorrhea decreased in both group, patients that took DNG showed a more rapid reduction compared with women that took EE/DNG, DNG treatment works faster than EE/DNG over time in symptom reduction, so, according to the literature, DNG was the best choice in the treatment of dysmenorrhea over time. Techatraisak [32] showed that DNG already improved scores for all EHP-30 scales at month 6 of treatment, until month 24. Caruso [19] considered that DNG was well tolerated with a favorable safety profile until a period of 65 weeks.

Women typically ask how the treatment will improve their symptoms, but they also want to know the possible side effects and it is well know that these can lead to a dropout of the therapy. So, in clinical practice, these are important informational ones to share with patients, counseling patients regarding the expected side effects, weighing up the efficacy and safety of each treatment approach [17].

We agree with the principles of evidence-based medicine that claims that effective and safe medical management of disease is a major clinical aim for the patients with endometriosis. The therapy must be personalized for each patient and also depends on the woman’s goal [12], considering pain as an important problem affecting the QoL of women [33]. As suggested by the NICE guidelines [34], a multidisciplinary approach, including psychologist and a patient association representative, is necessary due to the complexity of the disease and its impact on psychological wellbeing too.

### Study limitations and strengths

As any observational study, this clinical experience has a huge risk of bias.

A limitation of the present study was the small sample size. Variables like endometrioma volume need a larger group. Only a small number had endometrioma, so we could not insert the size of endometrioma as a dependent variable. In further research, it will be interesting to study if DNG improves QoL, HRQoL and symptoms over time regardless to the reduction of endometrioma size.

Moreover, a better understanding of all the bio-psycho-social aspects involved in women’s wellbeing and pain experience regarding endometriosis needs further research in longer periods.

The side effect profile was collected in the routine clinical practice and not systematically studied. Failure to evaluation for this variable may have introduced a confounding bias. In further randomized research, it will be important to study the relation between effect sides and QoL patients other than the risk of dropout of the therapy.

Strengths of our study were strict inclusion criteria and accuracy in choice of different measures to assess psychological wellness. Another advantage is the cooperation among different qualified researchers with high experience.

## Conclusion

The results of this study highlight that endometriosis causes a general worsening in many areas of the QoL of patients suffering from it. In particular, women with endometriosis show both poor physical and mental components of QoL. Pain perception is associated with worsening of different components of generic QoL, specific HRQoL and sexual problems. DUE is related to worsening of different component of both QoL and HRQoL. As concerns the effect of treatment over time, the current findings demonstrate an improvement in the physical component of QoL, in general pain perception, in different areas of HRQoL (emotional wellbeing, relationship with the medical profession) and in symptoms (dysmenorrhea) using both DNG and EE/DNG. Moreover, DNG seems to have a greater effect than EE/DNG on dyspareunia reduction over time. Consequently, as well as improvement in pain symptoms, an improvement in QoL is a crucial aspect in endometriosis toward a global management of the disease.

The results of this study, highlight the need for multidisciplinary approach and clinician education to decrease the effect of endometriosis on women’s QoL and to reduce the duration of untreated endometriosis.

## Data Availability

The datasets generated during and/or analysed during the current study are not publicly available due to privacy policy but are available from the corresponding author on reasonable request.

## Abbreviations

PCS: Physical Component summary score
MCS: Mental Component summary score
DUE: Duration untreated endometriosis
DNG: Dienogest 2 mg/daily
EE/DNG: Dienogest/ethinylestradiol 0.03 mg/daily
QoL: Quality of Life
HRQoL: Health-Related Quality of Life
T0: Time 0
T1: Time 1
VAS: Visual Analogic Scale
SF-36: Short Form-36
EHP-30: Endometriosis Health Profile-30
ISS: Index of sexual satisfaction
